# The serotonin theory of depression: a systematic umbrella review of the evidence

**DOI:** 10.1038/s41380-022-01661-0

**Published:** 2022-07-20

**Authors:** Joanna Moncrieff, Ruth E. Cooper, Tom Stockmann, Simone Amendola, Michael P. Hengartner, Mark A. Horowitz

**Affiliations:** 1 Division of Psychiatry, University College London, London, UK; 2 Research and Development Department, Goodmayes Hospital, North East London NHS Foundation Trust, Essex, UK; 3 Faculty of Education, Health and Human Sciences, University of Greenwich, London, UK; 4 Psychiatry-UK, Cornwall, UK; 5 Department of Dynamic and Clinical Psychology, and Health Studies, Faculty of Medicine and Psychology, Sapienza University of Rome, Rome, Italy; 6 Department of Applied Psychology, Zurich University of Applied Sciences, Zurich, Switzerland

**Keywords:** Diagnostic markers, Depression

## Abstract

The serotonin hypothesis of depression is still influential. We aimed to synthesise and evaluate evidence on whether depression is associated with lowered serotonin concentration or activity in a systematic umbrella review of the principal relevant areas of research. PubMed, EMBASE and PsycINFO were searched using terms appropriate to each area of research, from their inception until December 2020. Systematic reviews, meta-analyses and large data-set analyses in the following areas were identified: serotonin and serotonin metabolite, 5-HIAA, concentrations in body fluids; serotonin 5-HT_1A_ receptor binding; serotonin transporter (SERT) levels measured by imaging or at post-mortem; tryptophan depletion studies; SERT gene associations and SERT gene-environment interactions. Studies of depression associated with physical conditions and specific subtypes of depression (e.g. bipolar depression) were excluded. Two independent reviewers extracted the data and assessed the quality of included studies using the AMSTAR-2, an adapted AMSTAR-2, or the STREGA for a large genetic study. The certainty of study results was assessed using a modified version of the GRADE. We did not synthesise results of individual meta-analyses because they included overlapping studies. The review was registered with PROSPERO (CRD42020207203). 17 studies were included: 12 systematic reviews and meta-analyses, 1 collaborative meta-analysis, 1 meta-analysis of large cohort studies, 1 systematic review and narrative synthesis, 1 genetic association study and 1 umbrella review. Quality of reviews was variable with some genetic studies of high quality. Two meta-analyses of overlapping studies examining the serotonin metabolite, 5-HIAA, showed no association with depression (largest *n* = 1002). One meta-analysis of cohort studies of plasma serotonin showed no relationship with depression, and evidence that lowered serotonin concentration was associated with antidepressant use (*n* = 1869). Two meta-analyses of overlapping studies examining the 5-HT_1A_ receptor (largest *n* = 561), and three meta-analyses of overlapping studies examining SERT binding (largest *n* = 1845) showed weak and inconsistent evidence of reduced binding in some areas, which would be consistent with increased synaptic availability of serotonin in people with depression, if this was the original, causal abnormaly. However, effects of prior antidepressant use were not reliably excluded. One meta-analysis of tryptophan depletion studies found no effect in most healthy volunteers (*n* = 566), but weak evidence of an effect in those with a family history of depression (*n* = 75). Another systematic review (*n* = 342) and a sample of ten subsequent studies (*n* = 407) found no effect in volunteers. No systematic review of tryptophan depletion studies has been performed since 2007. The two largest and highest quality studies of the SERT gene, one genetic association study (*n* = 115,257) and one collaborative meta-analysis (*n* = 43,165), revealed no evidence of an association with depression, or of an interaction between genotype, stress and depression. The main areas of serotonin research provide no consistent evidence of there being an association between serotonin and depression, and no support for the hypothesis that depression is caused by lowered serotonin activity or concentrations. Some evidence was consistent with the possibility that long-term antidepressant use reduces serotonin concentration.

## Introduction

The idea that depression is the result of abnormalities in brain chemicals, particularly serotonin (5-hydroxytryptamine or 5-HT), has been influential for decades, and provides an important justification for the use of antidepressants. A link between lowered serotonin and depression was first suggested in the 1960s [1], and widely publicised from the 1990s with the advent of the Selective Serotonin Reuptake Inhibitor (SSRI) antidepressants [2–4]. Although it has been questioned more recently [5, 6], the serotonin theory of depression remains influential, with principal English language textbooks still giving it qualified support [7, 8], leading researchers endorsing it [9–11], and much empirical research based on it [11–14]. Surveys suggest that 80% or more of the general public now believe it is established that depression is caused by a ‘chemical imbalance’ [15, 16]. Many general practitioners also subscribe to this view [17] and popular websites commonly cite the theory [18].

It is often assumed that the effects of antidepressants demonstrate that depression must be at least partially caused by a brain-based chemical abnormality, and that the apparent efficacy of SSRIs shows that serotonin is implicated. Other explanations for the effects of antidepressants have been put forward, however, including the idea that they work via an amplified placebo effect or through their ability to restrict or blunt emotions in general [19, 20].

Despite the fact that the serotonin theory of depression has been so influential, no comprehensive review has yet synthesised the relevant evidence. We conducted an ‘umbrella’ review of the principal areas of relevant research, following the model of a similar review examining prospective biomarkers of major depressive disorder [21]. We sought to establish whether the current evidence supports a role for serotonin in the aetiology of depression, and specifically whether depression is associated with indications of lowered serotonin concentrations or activity.

## Methods

### Search strategy and selection criteria

The present umbrella review was reported in accordance with the 2009 PRISMA statement [22]. The protocol was registered with PROSPERO in December 2020 (registration number CRD42020207203) (https://www.crd.york.ac.uk/prospero/display_record.php?RecordID=207203). This was subsequently updated to reflect our decision to modify the quality rating system for some studies to more appropriately appraise their quality, and to include a modified GRADE to assess the overall certainty of the findings in each category of the umbrella review.

In order to cover the different areas and to manage the large volume of research that has been conducted on the serotonin system, we conducted an ‘umbrella’ review. Umbrella reviews survey existing systematic reviews and meta-analyses relevant to a research question and represent one of the highest levels of evidence synthesis available [23]. Although they are traditionally restricted to systematic reviews and meta-analyses, we aimed to identify the best evidence available. Therefore, we also included some large studies that combined data from individual studies but did not employ conventional systematic review methods, and one large genetic study. The latter used nationwide databases to capture more individuals than entire meta-analyses, so is likely to provide even more reliable evidence than syntheses of individual studies.

We first conducted a scoping review to identify areas of research consistently held to provide support for the serotonin hypothesis of depression. Six areas were identified, addressing the following questions: (1) Serotonin and the serotonin metabolite 5-HIAA–whether there are lower levels of serotonin and 5-HIAA in body fluids in depression; (2) Receptors - whether serotonin receptor levels are altered in people with depression; (3) The serotonin transporter (SERT) - whether there are higher levels of the serotonin transporter in people with depression (which would lower synaptic levels of serotonin); (4) Depletion studies - whether tryptophan depletion (which lowers available serotonin) can induce depression; (5) SERT gene – whether there are higher levels of the serotonin transporter gene in people with depression; (6) Whether there is an interaction between the SERT gene and stress in depression.

We searched for systematic reviews, meta-analyses, and large database studies in these six areas in PubMed, EMBASE and PsycINFO using the Healthcare Databases Advanced Search tool provided by Health Education England and NICE (National Institute for Health and Care Excellence). Searches were conducted until December 2020.

We used the following terms in all searches: (depress* OR affective OR mood) AND (systematic OR meta-analysis), and limited searches to title and abstract, since not doing so produced numerous irrelevant hits. In addition, we used terms specific to each area of research (full details are provided in Table S1, Supplement). We also searched citations and consulted with experts.

Inclusion criteria were designed to identify the best available evidence in each research area and consisted of:

1. Research synthesis including systematic reviews, meta-analysis, umbrella reviews, individual patient meta-analysis and large dataset analysis.
2. Studies that involve people with depressive disorders or, for experimental studies (tryptophan depletion), those in which mood symptoms are measured as an outcome.
3. Studies of experimental procedures (tryptophan depletion) involving a sham or control condition.
4. Studies published in full in peer reviewed literature.
5. Where more than five systematic reviews or large analyses exist, the most recent five are included.

Exclusion criteria consisted of:

1. Animal studies.
2. Studies exclusively concerned with depression in physical conditions (e.g. post stroke or Parkinson’s disease) or exclusively focusing on specific subtypes of depression such as postpartum depression, depression in children, or depression in bipolar disorder.

No language or date restrictions were applied. In areas in which no systematic review or meta-analysis had been done within the last 10 years, we also selected the ten most recent studies at the time of searching (December 2020) for illustration of more recent findings. We performed this search using the same search string for this domain, without restricting it to systematic reviews and meta-analyses.

### Data analysis

Each member of the team was allocated one to three domains of serotonin research to search and screen for eligible studies using abstract and full text review. In case of uncertainty, the entire team discussed eligibility to reach consensus.

For included studies, data were extracted by two reviewers working independently, and disagreement was resolved by consensus. Authors of papers were contacted for clarification when data was missing or unclear.

We extracted summary effects, confidence intervals and measures of statistical significance where these were reported, and, where relevant, we extracted data on heterogeneity. For summary effects in the non-genetic studies, preference was given to the extraction and reporting of effect sizes. Mean differences were converted to effect sizes where appropriate data were available.

We did not perform a meta-analysis of the individual meta-analyses in each area because they included overlapping studies [24]. All extracted data is presented in Table 1. Sensitivity analyses were reported where they had substantial bearing on interpretation of findings.

**Table 1:** Study characteristics and results.

Study	Design	*N* total: studies, participants (cases; controls where relevant)	Serotonin measure	Medication status	Summary effect (95% CI), *p* Heterogeneity-*p*, *I*^*2*^ (95% CI), N for analysis (if different from total sample size)	Quality rating (AMSTAR-2/ *STREGA) % satisfactory (of applicable terms)
**Serotonin and 5HIAA:** case (depression) vs. healthy control studies comparing concentration of serotonin in plasma and CSF						
Huang et al., 2020	Meta-analysis of cohort studies	3 studies, 663 women with depression; 1806 controls	5-HT in plasma	18.3% of patients were taking antidepressants.	*β* = −0.26 (−0.48 to −0.03), *p* > 0.05 after correction for multiple testing Heterogeneity not reported Sensitivity analysis: No difference in levels of 5-HT in women with depression not taking antidepressants compared to controls (women without depression not taking antidepressants) (*p* = 0.528) Lower levels of 5-HT in women with depression taking antidepressants compared to controls (*p* < 0.0001) and in women taking antidepressants without depression compared to controls (*p* < 0.0001)	60%
Ogawa et al., 2018	Systematic review and meta-analysis	11 studies, 435 people with depression; 380 controls	5-HIAA in CSF	All participants drug free – washout: 4–57 days (mode 14 days)	*g* = 0.042 (−0.26 to 0.17), *p* = 0.70, heterogeneity: I^2^ = 48.24 (*p* = 0.026)	48%
Pech et al., 2018	Systematic review and meta-analysis	13 studies, 529 people with depression; 473 controls	5-HIAA in CSF	10 studies: patients were drug free - washout: 4 days-6 weeks (mostly 2–4 weeks). 2 studies: some patients used occasional antidepressants. 1 study: majority of patients taking imipramine and chlorpromazine	Mean difference = −3.85 (−8.89 to 1.19) (*g* = 0.06), *p* = 0.14, heterogeneity – not reported	38%
**Receptors:** case (depression) vs. healthy control studies comparing 5-HT_**1**_ receptor binding						
Nikolaus et al., 2016	Systematic review and meta-analysis	14 studies, 245 people with depression; 316 controls	5-HT_**1A**_ receptor binding	2 studies conducted with drug naïve patients (total *N* = 19). 11 studies - prior treatment with antidepressants and/or other psychiatric drugs – washout: 1–1825 days (mode 14 days) 1 study medication not specified	Trend for increased 5-HT_**1**_ in parahippocampal gyrus (+23%), *p* = 0.096. Trend for reduced 5-HT_**1**_ in midbrain (−17%), *p* = 0.076. *N* and heterogeneity – not reported for any brain areas.	19%
Wang et al., 2016	Systematic review and meta-analysis	10 studies, 218 people with depression; 261 controls	5-HT_**1A**_ receptor binding	9 studies: drug-free – washout: 2–26 weeks (mainly 2–3 weeks). 1 study: medication naïve.	Reduced 5-HT_1A_ binding mesiotemporal cortex SMD = −0.8 (−1.36, −0.24), *p* = 0.005, heterogeneity: I^2^ = 87% (*P* < 0.00001) 218 patients, 261 controls** Reduced 5-HT_1A_ binding hippocampus SMD = −0.29 (−0.51, −0.07), *P* = 0.010, heterogeneity: I^2^ = 33%, *p* = 0.18 148 patients, 203 controls. Reduced 5-HT_1A_ binding raphe nucleus SMD = −0.60 (−1.17, −0.04), *p* = 0.04 Heterogeneity: I^2^ = 88%, *p* < 0.00001 218 patients, 261 controls Reduced 5-HT_1A_ binding insular cortex SMD = −0.79 (−1.54, to −0.05), *p* = 0.04. Heterogeneity: I^2^ = 91%, *p* < 0.00001 180 patients, 225 controls. There was no significant difference in the occipital cortex and the anterior cingulate cortex.	52%
**Serotonin transporter (SERT):** case (depression) vs. healthy control studies comparing SERT binding						
Nikolaus et al., 2016	Systematic review and meta-analysis	35 studies, 694 people with depression; 700 controls	SERT binding	26 studies: drug-free - washout: 3–900 days. 5 studies: drug naive 2 studies: patients medicated when scanned. 2 studies: did not report medication.	Reduced SERT in thalamus −12%, *p* = 0.004 Reduced SERT in amygdala −15%, *p* = 0.04. Reduced SERT in midbrain/ pons −8%, *p* = 0.005 Increased SERT in insula +9%, *p* = 0.037 *N*, CI and heterogeneity – not reported for any brain areas.	19%
Kambeitz & Howes., 2015	Systematic review and meta-analysis	25 in vivo imaging studies, 25 post-mortem studies, 877 people with depression; 968 controls	SERT binding	In vivo studies: three studies – antidepressant naïve patients. Post Mortem studies: medication status not reported	**In vivo** Reduced SERT in brainstem *g* = −0.31 (−0.55 to −0.08), *p* = 0.01, heterogeneity - I^2^ = 60.69% (95% CI: 34.39 to 83.35%), *N* = 880 Reduced SERT in midbrain *g* = −0.28 (−0.49 to −0.07), *p* = 0.01, heterogeneity - I^2^ = 49.68% (95% CI: 14.34% to 78.72%), *N* = 827 Reduced SERT in amygdala *g* = −0.37*** (−0.61 to −0.13), p<0.01, heterogeneity - I^2^ = 0% (95%-CI: 0% to 75.38%), *N* = 318 Reduced SERT in striatum *g* = −0.39 (−0.62 to −0.17), P<.001, heterogeneity - I^2^ = 6.7% (0% to 78.1%), *N* = 370 No difference: hippocampus, thalamus, cingulate cortex, frontal cortex. **Post-mortem:** Reduced SERT in hippocampus *g* = −0.63 (−1.12 to −0.15), *p* = 0.01 heterogeneity - I^2^ = 43.97% (0% to 93.34), *N* = 141 Non-significant after correction for publication bias; *g* = 0.32, *p* = 0.32 No difference: brainstem, frontal lobe. Meta-analysis not possible for other areas	48%
Gryglewski et al., 2014	Systematic review and meta-analysis	18 studies, 364 people with depression; 372 controls	SERT binding	149/364 patients - drug naïve; others drug free: 5 days to > 1 year (median 7 weeks)	Reduced SERT in midbrain: *g* = −0.49 (−0.84 to −0.14), heterogeneity - I^2^ = 68.7%, 313 patients, 321 controls. Reduced SERT in amygdala *g* = −0.50 (−0.78 to −0.22), heterogeneity - I^2^ = 0%, 96 patients, 128 controls. No difference: brainstem, thalamus, striatum, frontal cortex, cingulate cortex.	48%
**Tryptophan depletion studies:** effect of acute tryptophan depletion (ATD) on mood in healthy volunteers; healthy volunteers with family history of depression; drug-free patients with MDD in remission						
Ruhé et al., 2007	Systematic review and meta-analysis	Included in meta-analysis: 32 healthy volunteer studies, *N* = 566; 19 patient studies *N* = 322	ATD	6 studies involved patients with prior use of antidepressants: 1 study: <3 months; 2 studies 1–3 months 3 studies >6 months 7 studies involved patients with current use of antidepressants. Remainder unspecified.	No effect of ATD in healthy volunteers in parallel group studies (negative for family history of depression): Pooled *g* = −0.63 (−1.95 to 0.70), *N* = 151. Sensitivity analysis excluding an outlier study reduced Hedges *g* to 0.16 (−0.43 to 0.76), *N* = 125. No effect of ATD in cross-over studies with volunteers with no family history of depression, *g* = −0.19 (−0.43 to 0.05), *p* = 0.13, heterogeneity - I^2^ = 65.6%, *p* < 0.001, *N* = 259^#^ ATD lowered mood in cross-over studies with healthy controls with family history of depression: Hedge’s *g* = −0.56 (−1.00 to −0.13), *p* = 0.01, heterogeneity - I^2^ = 50.8%, *p* = 0.06, *N* = 75. ATD lowered mood in cross-over studies in drug-free people with remitted MDD: *g* = −1.90 (−3.02 to −0.78), *p* = 0.0009, heterogeneity - I^2^ = 89.4%, P< 0.00001, *N* = 97. ATD lowered mood in cross-over studies with people with remitted MDD currently using antidepressants : *g* = −0.49 (−0.89 to −0.10), *p* = 0.01, heterogeneity - I^2^ = 53.3%, *P* = 0.04, *N* = 83.	71%
Fusar-Poli et al., 2006	Systematic review and narrative synthesis	22 studies (23 cohorts), 64 people with remitted depression; 278 controls	ATD	A portion of the remitted depressed group were taking antidepressants but exact proportion not reported	Results reported narratively. 17/19 studies involving healthy volunteers showed no effect of tryptophan depletion on mood. 4 studies in patients with remitted depression, an unspecified portion of whom were taking antidepressants, found a decrease in mood following tryptophan depletion. Effect sizes or statistical significance were not reported.	22%
**SERT gene:** association between SERT gene and depression						
Border et al., 2019	Genetic association study	Data from two genetic data banks, 48,190–115,257 individuals	Association between 5-HTTLPR polymorphism and depression	N/A	No relationship between 5-HTTLPR polymorphism and estimated lifetime MDD diagnosis OR = 1.000, *p* = 0.994 (*N* = 115,257) None of seven other depression outcomes (e.g. estimated lifetime diagnosis, current depression severity) showed an association with the 5-HTTLPR polymorphism. *N* = 48,190–115,257	88%*
Culverhouse et al., 2018	Collaborative meta-analysis	31 data-sets, 43,165 Individuals	5-HTTLPR polymorphism association with depression	N/A	No significant effect of number of copies of s-allele of 5HTTLPR on lifetime depression. OR 1.00 (0.95 to 1.05), *p* = 0.95, *N* = 21,135. Heterogeneity- not reported All other analyses with variations of stress exposure and depression evaluation were non-significant with OR 1.00–1.08, *p* = 0.36–0.97, *N* = 13,835–28,252	100%
Oo et al., 2016	Systematic review and meta-analysis	23 studies, 3392 people with depression; 5093 controls	5-HTTLPR polymorphism association with depression	N/A	5-HTTLPR polymorphism associated with depression (per S allele): OR = 1.16 (1.08 to 1.23), *p*-value. Heterogeneity- I^2^ = 29.3%, *p* = 0.09 Homozygote carriers of the S allele of 5HTTLPR polymorphism compared with heterozygote and non-carriers combined (SS vs SL+LL genotype): OR = 1.33 (1.19 to 1.48) for major depressive disorder. Heterogeneity- I^2^ = 0.1%, *p* = 0.46	62%
Gatt et al., 2015	Umbrella review	11 meta-analyses included 1014–14,250 participants	7 meta-analyses of case control studies of 5-HTTLPR polymorphism and depression. 4 meta-analyses of GWAS	N/A	Individual gene meta-analyses: 3 studies found the S allele or SS genotype associated with depression in mixed and Caucasian samples (OR 1.11 (1.04–1.19), N = 9459 OR 1.23 (1.01–1.52), *N* = 1014 OR 1.40 (1.19–1.65), *N* = 6884) 3 studies reported no effect of the S allele in mixed and Caucasian samples 2 studies reported no effect of the SS genotype in Caucasian samples 3 studies found that the S allele or SS genotype had no effect in Asian samples No evidence of association in all 4 GWAS meta-analyses (*n* = 6566 to *n* = 12,664)	17%
Kiyohara & Yoshimasu, 2010	Systematic review and meta-analysis	22 studies, 2934 people with depression; 4985 controls	5-HTTLPR polymorphism association with depression	N/A	5-HTTLPR polymorphism (SS vs LL) associated with depression in Caucasian samples (but not Asian samples): OR = 1.41 (1.15 to 1.72), Heterogeneity *p* = 0.23 (*N* = 5756)	57%
**Gene-stress interaction**						
Border et al., 2019	See Border et al., 2019 (above)	See Border et al., 2019 (above)	5-HTTLPR Polymorphism x environmental interaction effect on depression	N/A	No significant interaction between the 5-HTTLPR polymorphism and childhood trauma. Exp(*β*) = 0.998, *p* = 0.919, *N* = 115,249. Heterogeneity not given as not a meta-analysis. 31 other analyses using different measures of stress or depression found no significant interaction.	See Border et al., 2019 (above)
Bleys et al 2018	Systematic review and meta-analysis	51 studies, *N* = 51,449	Interaction between stress and 5-HTTLPR polymorphism in depression	N/A	OR = 1.18 (1.09 to 1.28). Heterogeneity- I^2^ = 52.4%, *p* < 0.0001	33%
Culverhouse et al., 2018	See Culverhouse et al., 2018 (above)	See Culverhouse et al., 2018 (above)	Interaction between stressful life events and 5-HTTLPR polymorphism for risk of depression	N/A	No significant interaction between number of copies of the s-allele for 5-HTTLPR and stress on risk of depression. OR = 1.05 (0.91 to 1.21), *p* = 0.49, *N* = 21,135 I^2^ = 0.0, *p* = 0.69 All other analyses with variations of stress and depression evaluation (including continuous measures of stress) were non-significant, with ORs from 0.85 to 1.06, *p* = 0.17–0.50, *N* = 13,835-28,252 I^2^ = 0–16.7, *p* = 0.26-0.87	See Culverhouse et al., 2018 (above)
Sharpley et al 2014	Systematic review and meta-analysis	81 studies, *N* = 55,269	Interaction between stress and 5-HTTLPR polymorphism in depression	N/A	OR not reported - Liptak-Stouffer Z-score method was used which combined p-values across studies, *p* = 9 × 10^−7^ Heterogeneity- not reported (*N* = 55,269)	48%
Karg et al 2011	Systematic review and meta-analysis	54 studies, *N* = 40,749	Whether the 5-HTTLPR polymorphism moderates the relationship between stress and depression	N/A	OR not reported - Liptak-Stouffer Z-score method was used which combined p-values across studies, *p* = 0.00002. Heterogeneity- not reported (*N* = 40,749)	29%

Extracted summary effects, confidence intervals and measures of statistical significance are reported. In reviews in which multiple meta-analyses were conducted, only summary effect sizes that were statistically significant are reported, with relevant negative findings that did not reach statistical significance listed. Where no relevant analysis was statistically significant, the results of the principal analysis (e.g., main gene effect) is reported. Data on heterogeneity is reported where relevant. Where sample size for a particular analysis was different from the total sample size, this is reported. For summary effects in the non-genetic studies, preference was given to the extraction and reporting of effect sizes. Mean differences were converted to effect sizes where appropriate data were available [25]. Where these data were not available the most relevant measure, such as the beta co-efficient, or average percentage change in ligand binding was reported. For genetic association studies, and gene-stress interactions studies we odds ratios are reported.

*CSF* Cerebrospinal fluid, *g* Hedges g, *GWAS* Genome Wide Association Studies, *MDD* Major Depressive Disorder, *N/A* not applicable, *OR* Odds Ratio, *SMD* Standard Mean Difference.

*STREGA rating; **where there were internal discrepancies between reported numbers in text or figures we obtained data from the original authors; ***where there were internal discrepancies between reported numbers in the text, and authors did not respond to queries, details from figures were prioritised, and statistics calculated from other data.

^#^sample size has a different meaning in crossover studies since each participant contributes data twice.

The quality rating of systematic reviews and meta-analyses was assessed using AMSTAR-2 (A MeaSurement Tool to Assess systematic Reviews) [25]. For two studies that did not employ conventional systematic review methods [26, 27] we used a modified version of the AMSTAR-2 (see Table S3). For the genetic association study based on a large database analysis we used the STREGA assessment (STrengthening the REporting of Genetic Association Studies) (Table S4) [28]. Each study was rated independently by at least two authors. We report ratings of individual items on the relevant measure, and the percentage of items that were adequately addressed by each study (Table 1, with further detail in Tables S3 and S4).

Alongside quality ratings, two team members (JM, MAH) rated the certainty of the results of each study using a modified version of the GRADE guidelines [29]. Following the approach of Kennis et al. [21], we devised six criteria relevant to the included studies: whether a unified analysis was conducted on original data; whether confounding by antidepressant use was adequately addressed; whether outcomes were pre-specified; whether results were consistent or heterogeneity was adequately addressed if present; whether there was a likelihood of publication bias; and sample size. The importance of confounding by effects of current or past antidepressant use has been highlighted in several studies [30, 31]. The results of each study were scored 1 or 0 according to whether they fulfilled each criteria, and based on these ratings an overall judgement was made about the certainty of evidence across studies in each of the six areas of research examined. The certainty of each study was based on an algorithm that prioritised sample size and uniform analysis using original data (explained more fully in the supplementary material), following suggestions that these are the key aspects of reliability [27, 32]. An assessment of the overall certainty of each domain of research examining the role of serotonin was determined by consensus of at least two authors and a direction of effect indicated.

## Results

### Search results and quality rating

**Fig. 1:**
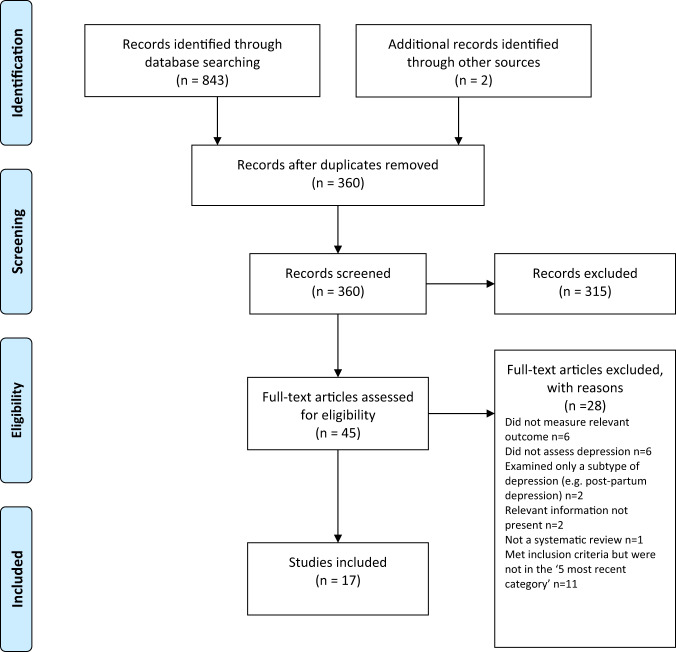
Preferred Reporting Items for Systematic reviews and Meta-Analyses (PRISMA) flow diagramme.

Searching identified 361 publications across the 6 different areas of research, among which seventeen studies fulfilled inclusion criteria (see Fig. 1 and Table S1 for details of the selection process). Included studies, their characteristics and results are shown in Table 1. As no systematic review or meta-analysis had been performed within the last 10 years on serotonin depletion, we also identified the 10 latest studies for illustration of more recent research findings (Table 2).

**Table 2:** Recent depletion studies comparing acute tryptophan depletion drink with amino acid balance drink (sham drink) - characteristics and results.

Study	Randomised controlled trial design	Participants: Healthy volunteers (HV) or people with depression*	Medication status	Summary effect (95% CI), *p*
Frey & McCabe, 2020	Parallel group, double blind	46 HV	No psychotropic medication for at least 3 months	No effect
Bar et al., 2020	Crossover trial, double blind	30 HV	Not specified	No effect
Deza-Araujo et al., 2018	Crossover trial, double blind	85 HV	Not specified	No effect
Martin et al., 2017	Crossover trial, double blind	15 HV	No history of neurological or psychiatric disorders	No effect
Eisner et al., 2016	Crossover trial, double blind	64 HV	No history of neurological or psychiatric disorders	No effect
Trotter et al., 2016	Parallel group, double blind	30 HV	Not specified	No effect
Hogenelst 2016	Crossover trial, double blind	40 HV with or without a family history of MDD	‘No use of psychotropics’	No effect
Weinstein et al., 2015	Crossover trial, double blind	15 people with depression	Treated with sertraline for 12 weeks prior to study	No effect
Moreno et al., 2015	Crossover trial, double blind	64 people with personal and family history of MDD; in remission	No ‘psychotropic medication’ for at least 3 months prior to test	A significant main effect of test condition (χ 2 = 5.14, d.f. = 1, *p* = 0.023) mainly due to improvement of HDRS scores following sham drink
Hembold et al., 2013	Crossover trial, double blind	18 HV	Not specified	No effect

*MDD* Major Depressive disorder, *HDRS* Hamilton depression rating scale.

*sample size has a different meaning in crossover studies since each participant contributes data twice.

Quality ratings are summarised in Table 1 and reported in detail in Tables S2–S3. The majority (11/17) of systematic reviews and meta-analyses satisfied less than 50% of criteria. Only 31% adequately assessed risk of bias in individual studies (a further 44% partially assessed this), and only 50% adequately accounted for risk of bias when interpreting the results of the review. One collaborative meta-analysis of genetic studies was considered to be of high quality due to the inclusion of several measures to ensure consistency and reliability [27]. The large genetic analysis of the effect of SERT polymorphisms on depression, satisfied 88% of the STREGA quality criteria [32].

### Serotonin and 5-HIAA

Serotonin can be measured in blood, plasma, urine and CSF, but it is rapidly metabolised to 5-hydroxyindoleacetic acid (5-HIAA). CSF is thought to be the ideal resource for the study of biomarkers of putative brain diseases, since it is in contact with brain interstitial fluid [33]. However, collecting CSF samples is invasive and carries some risk, hence large-scale studies are scarce.

Three studies fulfilled inclusion criteria (Table 1). One meta-analysis of three large observational cohort studies of post-menopausal women, revealed lower levels of plasma 5-HT in women with depression, which did not, however, reach statistical significance of *p* < 0.05 after adjusting for multiple comparisons. Sensitivity analyses revealed that antidepressants were strongly associated with lower serotonin levels independently of depression.

Two meta-analyses of a total of 19 studies of 5-HIAA in CSF (seven studies were included in both) found no evidence of an association between 5-HIAA concentrations and depression.

### Receptors

Fourteen different serotonin receptors have been identified, with most research on depression focusing on the 5-HT_**1A**_ receptor [11, 34]. Since the functions of other 5-HT receptors and their relationship to depression have not been well characterised, we restricted our analysis to data on 5-HT_**1A**_ receptors [11, 34]. 5-HT_**1A**_ receptors, known as auto-receptors, inhibit the release of serotonin pre-synaptically [35], therefore, if depression is the result of reduced serotonin activity caused by abnormalities in the 5-HT_**1A**_ receptor, people with depression would be expected to show increased activity of 5-HT_**1A**_ receptors compared to those without [36].

Two meta-analyses satisfied inclusion criteria, involving five of the same studies [37, 38] (see Table 1). The majority of results across the two analyses suggested either no difference in 5-HT_1A_ receptors between people with depression and controls, or a lower level of these inhibitory receptors, which would imply higher concentrations or activity of serotonin in people with depression. Both meta-analyses were based on studies that predominantly involved patients who were taking or had recently taken (within 1–3 weeks of scanning) antidepressants or other types of psychiatric medication, and both sets of authors commented on the possible influence of prior or current medication on findings. In addition, one analysis was of very low quality [37], including not reporting on the numbers involved in each analysis and using one-sided p-values, and one was strongly influenced by three studies and publication bias was present [38].

### The serotonin transporter (SERT)

The serotonin transporter protein (SERT) transports serotonin out of the synapse, thereby lowering the availability of serotonin in the synapse [39, 40]. Animals with an inactivated gene for SERT have higher levels of extra-cellular serotonin in the brain than normal [41–43] and SSRIs are thought to work by inhibiting the action of SERT, and thus increasing levels of serotonin in the synaptic cleft [44]. Although changes in SERT may be a marker for other abnormalities, if depression is caused by low serotonin availability or activity, and if SERT is the origin of that deficit, then the amount or activity of SERT would be expected to be higher in people with depression compared to those without [40]. SERT binding potential is an index of the concentration of the serotonin transporter protein and SERT concentrations can also be measured post-mortem.

Three overlapping meta-analyses based on a total of 40 individual studies fulfilled inclusion criteria (See Table 1) [37, 39, 45]. Overall, the data indicated possible reductions in SERT binding in some brain areas, although areas in which effects were detected were not consistent across the reviews. In addition, effects of antidepressants and other medication cannot be ruled out, since most included studies mainly or exclusively involved people who had a history of taking antidepressants or other psychiatric medications. Only one meta-analysis tested effects of antidepressants, and although results were not influenced by the percentage of drug-naïve patients in each study, numbers were small so it is unlikely that medication-related effects would have been reliably detected [45]. All three reviews cited evidence from animal studies that antidepressant treatment reduces SERT [46–48]. None of the analyses corrected for multiple testing, and one review was of very low quality [37]. If the results do represent a positive finding that is independent of medication, they would suggest that depression is associated with higher concentrations or activity of serotonin.

### Depletion studies

Tryptophan depletion using dietary means or chemicals, such as parachlorophenylalanine (PCPA), is thought to reduce serotonin levels. Since PCPA is potentially toxic, reversible tryptophan depletion using an amino acid drink that lacks tryptophan is the most commonly used method and is thought to affect serotonin within 5–7 h of ingestion. Questions remain, however, about whether either method reliably reduces brain serotonin, and about other effects including changes in brain nitrous oxide, cerebrovascular changes, reduced BDNF and amino acid imbalances that may be produced by the manipulations and might explain observed effects independent of possible changes in serotonin activity [49].

One meta-analysis and one systematic review fulfilled inclusion criteria (see Table 1). Data from studies involving volunteers mostly showed no effect, including a meta-analysis of parallel group studies [50]. In a small meta-analysis of within-subject studies involving 75 people with a positive family history, a minor effect was found, with people given the active depletion showing a larger decrease in mood than those who had a sham procedure [50]. Across both reviews, studies involving people diagnosed with depression showed slightly greater mood reduction following tryptophan depletion than sham treatment overall, but most participants had taken or were taking antidepressants and participant numbers were small [50, 51].

Since these research syntheses were conducted more than 10 years ago, we searched for a systematic sample of ten recently published studies (Table 2). Eight studies conducted with healthy volunteers showed no effects of tryptophan depletion on mood, including the only two parallel group studies. One study presented effects in people with and without a family history of depression, and no differences were apparent in either group [52]. Two cross-over studies involving people with depression and current or recent use of antidepressants showed no convincing effects of a depletion drink [53, 54], although one study is reported as positive mainly due to finding an improvement in mood in the group given the sham drink [54].

### SERT gene and gene-stress interactions

A possible link between depression and the repeat length polymorphism in the promoter region of the SERT gene (5-HTTLPR), specifically the presence of the short repeats version, which causes lower SERT mRNA expression, has been proposed [55]. Interestingly, lower levels of SERT would produce higher levels of synaptic serotonin. However, more recently, this hypothesis has been superseded by a focus on the interaction effect between this polymorphism, depression and stress, with the idea that the short version of the polymorphism may only give rise to depression in the presence of stressful life events [55, 56]. Unlike other areas of serotonin research, numerous systematic reviews and meta-analyses of genetic studies have been conducted, and most recently a very large analysis based on a sample from two genetic databanks. Details of the five most recent studies that have addressed the association between the SERT gene and depression, and the interaction effect are detailed in Table 1.

Although some earlier meta-analyses of case-control studies showed a statistically significant association between the 5-HTTLPR and depression in some ethnic groups [57, 58], two recent large, high quality studies did not find an association between the SERT gene polymorphism and depression [27, 32]. These two studies consist of  by far the largest and most comprehensive study to date [32] and a high-quality meta-analysis that involved a consistent re-analysis of primary data across all conducted studies, including previously unpublished data, and other comprehensive quality checks [27, 59] (see Table 1).

Similarly, early studies based on tens of thousands of participants suggested a statistically significant interaction between the SERT gene, forms of stress or maltreatment and depression [60–62], with a small odds ratio in the only study that reported this (1.18, 95% CI 1.09 to 1.28) [62]. However, the two recent large, high-quality studies did not find an interaction between the SERT gene and stress in depression (Border et al [32] and Culverhouse et al.) [27] (see Table 1).

### Overall results

Table 3 presents the modified GRADE ratings for each study and the overall rating of the strength of evidence in each area. Areas of research that provided moderate or high certainty of evidence such as the studies of plasma serotonin and metabolites and the genetic and gene-stress interaction studies all showed no association between markers of serotonin activity and depression. Some other areas suggested findings consistent with increased serotonin activity, but evidence was of very low certainty, mainly due to small sample sizes and possible residual confounding by current or past antidepressant use. One area - the tryptophan depletion studies - showed very low certainty evidence of lowered serotonin activity or availability in a subgroup of volunteers with a family history of depression. This evidence was considered very low certainty as it derived from a subgroup of within-subject studies, numbers were small, and there was no information on medication use, which may have influenced results. Subsequent research has not confirmed an effect with numerous negative studies in volunteers.

**Table 3:** Modified GRADE ratings for each study and the overall rating of strength of evidence.

Study	Unified statistical analysis on original data	Confounding by antidepressant use adequately excluded (where effect found)*	Outcomes of interest pre-specified	Consistent results (or heterogeneity adequately addressed if present)	Little likelihood of publication bias (in funnel plots or tests)	Large sample (>500 for non-genetic and >10,000 for genetic)	Certainty of evidence	Overall certainty of domain
Serotonin and 5HIAA: case (depression) vs. healthy control studies comparing concentration of serotonin in plasma and CSF								
Huang et al., 2020	1	1	1	0	1	1	Moderate certainty evidence of no effect (lowered serotonin explained by antidepressants)	Moderate certainty evidence of no effect
Ogawa et al., 2018	0	N/A	1	1	1	1	Moderate certainty evidence of no effect	
Pech et al., 2018	0	N/A	1	1	0	1	Low certainty evidence of no effect	
Receptors: case (depression) vs. healthy control studies comparing 5-HT_**1**_ receptor binding								
Nikolaus et al., 2016	0	0	0	0	0	0	Very low certainty evidence of increased serotonin activity	Very low certainty evidence of increased serotonin activity
Wang et al., 2016	0	0	1	0	0	0	Very low certainty evidence of increased serotonin activity	
Serotonin transporter (SERT): case (depression) vs. healthy control studies comparing SERT binding								
Nikolaus et al., 2016	0	0	0	0	0	0	Very low certainty evidence of increased serotonin activity	Very low certainty evidence of increased serotonin activity
Kambeitz & Howes., 2015	0	0	1	1	0	1	Very low certainty evidence of increased serotonin activity	
Gryglewski et al., 2014	0	0	1	1	0	1	Very low certainty evidence of increased serotonin activity	
Tryptophan depletion studies: effect of acute tryptophan depletion (ATD) on mood in healthy volunteers; healthy volunteers with family history of depression; drug-free patients with MDD in remission								
Ruhe et al., 2007	0	0	1	1	0	0	Very low certainty evidence of no effect for people with no family history of depression. Very low certainty evidence of lowered serotonin activity in those with a family history and people with depression in remission.	Very low certainty evidence of no effect or lowered serotonin activity in vulnerable populations
Fusar-Poli et al., 2006	0	N/A	1	0	0	0	Very low certainty evidence of lowered serotonin activity	
SERT gene: association between SERT gene and depression								
Border et al., 2019	1	N/A	1	N/A	1	1	High certainty evidence of no effect of the SERT gene	High certainty evidence of no effect for SERT gene
Culverhouse et al., 2018	1	N/A	1	1	1	1	High certainty evidence of no effect of the SERT gene	
Oo et al., 2016	0	N/A	0	1	1	0	Very low certainty evidence of an effect for SERT gene	
Gatt et al., 2015	0	N/A	0	0	0	0	Very low certainty evidence of an effect for SERT gene	
Kiyohara & Yoshimasu, 2010	0	N/A	1	0	1	0	Very low certainty evidence of an effect for SERT gene	
Gene-stress interaction								
Border et al., 2019	1	N/A	1	N/A	1	1	High certainty evidence of no effect for gene-stress interaction	High certainty evidence of no effect for gene-stress interaction
Bleys et al., 2018	0	N/A	1	0	1	1	Low certainty evidence of an effect for gene-stress interaction	
Culverhouse et al., 2018	1	N/A	1	1	1	1	High certainty evidence of no effect for gene-stress interaction	
Sharpley et al., 2014	0	N/A	1	0	1	1	Low certainty evidence of an effect for gene-stress interaction	
Karg et al., 2011	0	N/A	1	0	1	1	Low certainty evidence of an effect for gene-stress interaction	

*N/A* not applicable

*Where no effect found or where prior use of medication is not relevant (as in genetic studies), item was rated as ‘not applicable’.

## Discussion

Our comprehensive review of the major strands of research on serotonin shows there is no convincing evidence that depression is associated with, or caused by, lower serotonin concentrations or activity. Most studies found no evidence of reduced serotonin activity in people with depression compared to people without, and methods to reduce serotonin availability using tryptophan depletion do not consistently lower mood in volunteers. High quality, well-powered genetic studies effectively exclude an association between genotypes related to the serotonin system and depression, including a proposed interaction with stress. Weak evidence from some studies of serotonin 5-HT_1A_ receptors and levels of SERT points towards a possible association between increased serotonin activity and depression. However, these results are likely to be influenced by prior use of antidepressants and its effects on the serotonin system [30, 31]. The effects of tryptophan depletion in some cross-over studies involving people with depression may also be mediated by antidepressants, although these are not consistently found [63].

The chemical imbalance theory of depression is still put forward by professionals [17], and the serotonin theory, in particular, has formed the basis of a considerable research effort over the last few decades [14]. The general public widely believes that depression has been convincingly demonstrated to be the result of serotonin or other chemical abnormalities [15, 16], and this belief shapes how people understand their moods, leading to a pessimistic outlook on the outcome of depression and negative expectancies about the possibility of self-regulation of mood [64–66]. The idea that depression is the result of a chemical imbalance also influences decisions about whether to take or continue antidepressant medication and may discourage people from discontinuing treatment, potentially leading to lifelong dependence on these drugs [67, 68].

As with all research synthesis, the findings of this umbrella review are dependent on the quality of the included studies, and susceptible to their limitations. Most of the included studies were rated as low quality on the AMSTAR-2, but the GRADE approach suggested some findings were reasonably robust. Most of the non-genetic studies did not reliably exclude the potential effects of previous antidepressant use and were based on relatively small numbers of participants. The genetic studies, in particular, illustrate the importance of methodological rigour and sample size. Whereas some earlier, lower quality, mostly smaller studies produced marginally positive findings, these were not confirmed in better-conducted, larger and more recent studies [27, 32]. The identification of depression and assessment of confounders and interaction effects were limited by the data available in the original studies on which the included reviews and meta-analyses were based. Common methods such as the categorisation of continuous measures and application of linear models to non-linear data may have led to over-estimation or under-estimation of effects [69, 70], including the interaction between stress and the SERT gene. The latest systematic review of tryptophan depletion studies was conducted in 2007, and there has been considerable research produced since then. Hence, we provided a snapshot of the most recent evidence at the time of writing, but this area requires an up to date, comprehensive data synthesis. However, the recent studies were consistent with the earlier meta-analysis with little evidence for an effect of tryptophan depletion on mood.

Although umbrella reviews typically restrict themselves to systematic reviews and meta-analyses, we aimed to provide the most comprehensive possible overview. Therefore, we chose to include meta-analyses that did not involve a systematic review and a large genetic association study on the premise that these studies contribute important data on the question of whether the serotonin hypothesis of depression is supported. As a result, the AMSTAR-2 quality rating scale, designed to evaluate the quality of conventional systematic reviews, was not easily applicable to all studies and had to be modified or replaced in some cases.

One study in this review found that antidepressant use was associated with a reduction of plasma serotonin [26], and it is possible that the evidence for reductions in SERT density and 5-HT_1A_ receptors in some of the included imaging study reviews may reflect compensatory adaptations to serotonin-lowering effects of prior antidepressant use. Authors of one meta-analysis also highlighted evidence of 5-HIAA levels being reduced after long-term antidepressant treatment [71]. These findings suggest that in the long-term antidepressants might produce compensatory changes [72] that are opposite to their acute effects [73, 74]. Lowered serotonin availability has also been demonstrated in animal studies following prolonged antidepressant administration [75]. Further research is required to clarify the effects of different drugs on neurochemical systems, including the serotonin system, especially during and after long-term use, as well as the physical and psychological consequences of such effects.

This review suggests that the huge research effort based on the serotonin hypothesis has not produced convincing evidence of a biochemical basis to depression. This is consistent with research on many other biological markers [21]. We suggest it is time to acknowledge that the serotonin theory of depression is not empirically substantiated.

### Supplementary information

Supplementary Tables

## Data Availability

All extracted data is available in the paper and supplementary materials. Further information about the decision-making for each rating for categories of the AMSTAR-2 and STREGA are available on request.
