# Supplementary material for: The serotonin theory of depression: a systematic umbrella review of the evidence
Source: Mol Psychiatry. 2022 Jul 20;28(8):3243–56. doi: 10.1038/s41380-022-01661-0 (PMC10618090; doi:10.1038/s41380-022-01661-0)
Supplement: Supplementary file 1 — Supplementary Tables [file 41380_2022_1661_MOESM1_ESM.docx]

**Table S1**

Search terms and number of papers reviewed: the Healthcare Databases Advanced Search tool provided by Health Education England and NICE (National Institute for health and Care Excellence) was used to search PubMed, EMBASE and PsycINFO.

| **Search terms** | **Records identified (N)** | **Records screened after duplicates removed (N)** | **Full text articles assessed for eligibility (N)** | **Full-text articles excluded with reasons (N)** | **Included (N)** |
| --- | --- | --- | --- | --- | --- |
| **Serotonin** |  |  |  |  |  |
| (depress* OR affective OR mood) AND (5HT OR 5-HT OR serotonin) AND (plasma OR blood OR urine OR CSF OR "cerebrospinal fluid" OR "cerebro-spinal fluid") AND (assay OR concentration OR spectrometry) AND ((systematic [sb]) OR (meta-analysis[Title/Abstract])) | 150 | 6 | 8 | 7  N=5 did not measure serotonin  N=1 did not assess depression  N=1 studied post-partum depression only | 1 |
| **5HIAA** |  |  |  |  |  |
| ((5HIAA) OR (((serotonin) OR (monoamine*) OR (neurotransmitter)) AND ((metabolite))) AND ((depress*) OR (affective) OR (mood)) AND ((systematic [sb]) OR (meta-analysis)) [Title/Abstract]) | 32 | 27 | 8 | 6  N=5 Not depression  N=1 Did not measure 5HIAA | 2 |
| **Receptors** |  |  |  |  |  |
| (depress*[Title/Abstract] OR affective[Title/Abstract] OR mood[Title/Abstract]) AND ("positron-Emission tomography"[Title/Abstract] OR pet[Title/Abstract] OR tomography[Title/Abstract] OR "emission-computed"[Title/Abstract] OR "single-photon"[Title/Abstract] OR SPECT[Title/Abstract] OR "molecular imaging" [Title/Abstract] OR "molecular diagno*"[Title/Abstract]) AND ((receptor[Title/Abstract] AND serotonin[Title/Abstract]) OR 5-HT1A[Title/Abstract] OR 5-HT2A[Title/Abstract] OR 5-HT1B[Title/Abstract] OR "serotonin 1B receptor"[Title/Abstract] OR "serotonin 1A receptor"[Title/Abstract] OR "serotonin 2A receptor"[Title/Abstract]) AND (systematic[Title/Abstract] OR meta-analysis[Title/Abstract]) | 15 | 9 | 3 | 1  Relevant information not present | 2 |
| **Serotonin transporter (SERT)** |  |  |  |  |  |
| ((((depress* OR affective OR mood) AND (systematic OR meta analysis)) AND (SLC6A4 OR 5HTTLPR OR 5-HTTLPR OR "serotonin transporter" OR "monoamine transporter" OR SERT OR 5-HTT OR 5HTT OR transporter)) AND (post-mortem OR "post mortem" OR imaging OR neuroimaging OR neuro-imaging)).ti,ab | 46 | 46 | 3 | 0 | 3 |
| **Tryptophan depletion studies** |  |  |  |  |  |
| (((tryptophan OR serotonin OR monoamine) AND depletion) AND (depress* OR affective OR mood)) AND (systematic OR meta analysis).ti,ab | 42 | 30 | 3 | 1  Non- systematic review | 2 |
| **SERT Gene** |  |  |  |  |  |
| (((SLC6A4 OR 5HTTLPR OR 5-HTTLPR OR "serotonin transporter" OR "monoamine transporter" OR SERT OR 5-HTT OR 5HTT) AND (depress* OR affective OR mood)) AND (gene OR genetic OR expression)) AND (systematic OR "meta analysis" OR meta-analysis).ti,ab | 438 | 177 | 15 | 10    N=1 Relevant information not present  N=9 Met inclusion criteria but were not in the ‘5 most recent category’ (as specified in the protocol) | 5 |
| **Gene-stress interaction** |  |  |  |  |  |
| (((SLC6A4 OR 5HTTLPR OR 5-HTTLPR OR "serotonin transporter" OR "monoamine transporter" OR SERT OR 5-HTT OR 5HTT) AND (depress* OR affective OR mood)) AND (stress OR "stressful life event")) AND (systematic OR "meta analysis" OR meta-analysis).ti,ab | 122 | 68 | 8 | 3  N=1 Examined only a sub-type of depression  Met inclusion criteria but were not in the ‘5 most recent category’ (as specified in the protocol) n=2 | 5 |

See Figure 1 for overall PRISMA diagram, including overall numbers of paper. Please note (Border et al., 2019; Culverhouse et al., 2018) were included in both SERT gene and gene-stress interaction; 1 paper (Nikolaus et al., 2016) was included in both SERT gene and receptors, which is why some totals are three higher than those shown in the PRISMA diagram.

**Table S2:** summary of excluded full-text papers

| **Reference** | **Reason for exclusion** |
| --- | --- |
| **Serotonin** |  |
| Li M; Kwok MK; Fong SSM; Schooling CM. Effects of tryptophan, serotonin, and kynurenine on ischemic heart diseases and its risk factors: a Mendelian Randomization study. *European journal of clinical nutrition*; 2020, 74 (4), 613-621  Zhu G; Yin Y; Xiao CL; Mao RJ; Shi BH et al.  Serum DHEAS levels are associated with the development of depression. *Psychiatry research*; 2015, 229 (no. 1-2), 447-453  Ogawa S; Fujii T; Koga N; Hori H; Teraishi T et al.  Plasma L-tryptophan concentration in major depressive disorder: new data and meta-analysis.*The Journal of clinical psychiatry*; 2014; vol. 75 (no. 9); p. e906  Kaplan JR; Muldoon MF; Manuck SB; Mann JJ. Assessing the observed relationship between low cholesterol and violence-related mortality. Implications for suicide risk. *Annals of the New York Academy of Sciences*; 1997; vol. 836; p. 57-80  Carney RM; Freedland KE. Depression and coronary heart disease: more pieces of the puzzle. *The American journal of psychiatry*; 2007; vol. 164 (9); p. 1307-1309 | Did not measure serotonin (N=5) |
| Camilleri M. Serotonin in the gastrointestinal tract.  Source Current opinion in endocrinology, diabetes, and obesity; Feb 2009; vol. 16 (1); p. 53-59 | Did not assess depression (N=1) |
| Luo Y; He GP. Correlative analysis of postpartum depression. *Journal of Central South University.* Medical sciences; 2007; vol. 32 (3); p. 460-465 | Studied post-partum depression only (N=1) |
| **5-HIAA** |  |
| Menon JML; Nolten C; Achterberg EJM; Joosten RNJMA; Dematteis M et al. Brain Microdialysate Monoamines in Relation to Circadian Rhythms, Sleep, and Sleep Deprivation - a Systematic Review, Network Meta-analysis, and New Primary Data. *Journal of circadian rhythms*; 2019; 17, p. 1  Knorr U; Simonsen AH; Zetterberg H; Blennow K; Hasselbalch SG et al. Biomarkers in cerebrospinal fluid of patients with bipolar disorder versus healthy individuals: A systematic review. *European neuropsychopharmacology*; 2018; vol. 28 (7), 783-794  Kawi J; Lukkahatai N; Inouye J; Thomason D; Connelly K.  Effects of Exercise on Select Biomarkers and Associated Outcomes in Chronic Pain Conditions: Systematic Review. *Biological research for nursing*; 2016; 18 (2); 147-159  Camilleri M. Serotonin in the gastrointestinal tract. *Current opinion in endocrinology, diabetes, and obesity*; 2009; 16 (1); p. 53-59  Kawi J.; Lukkahatai N.; Inouye J.Thomason D.; Connelly K. Effects of Exercise on Select Biomarkers and Associated Outcomes in Chronic Pain Conditions: Systematic Review. *Biological research for nursing*; 2016; 18 (2); 147-159 | Not depression (N=5) |
| Ogyu K; Kubo K; Noda Y; Iwata Y; Tsugawa S et al. Kynurenine pathway in depression: A systematic review and meta-analysis. *Neuroscience and biobehavioral reviews;* 2018; 90, 16-25 | Did not measure 5-HIAA (N=1) |
| **Receptors** |  |
| Serotonin-1A receptors in major depression quantified using PET: controversies, confounds, and recommendations. Shrestha S; Hirvonen J; Hines CS; Henter ID; Svenningsson P et al. *NeuroImage*; 2012; 59 (4), 3243-3251 | Relevant information not present (N=1) |
| **Serotonin transporter (SERT)** |  |
| No exclusions |  |
| **Tryptophan depletion studies** |  |
| Bell, C., Abrams, J., & Nutt, D. (2001). Tryptophan depletion and its implications for psychiatry. *The British Journal of Psychiatry*, *178*(5), 399-405 | Non-systematic review (N=1) |
| **SERT Gene** |  |
| Lacerda-Pinheiro SF, Pinheiro Junior RF, Pereira de Lima MA, Lima da Silva CG, Vieira dos Santos Mdo S, Teixeira Júnior AG, Lima de Oliveira PN, Ribeiro KD, Rolim-Neto ML, Bianco BA. Are there depression and anxiety genetic markers and mutations? A systematic review*. J Affect Disord.* 2014, 168:387-98. | Relevant information not present (N=1) |
| Levinson DF. Meta-analysis in psychiatric genetics. *Curr Psychiatry Rep.* 2005, 7(2):143-51.  López-León, S., Janssens, A., González-Zuloeta Ladd, A. et al. Meta-analyses of genetic studies on major depressive disorder. *Mol Psychiatry,* 13, 772–785 (2008).  Anguelova M, Benkelfat C, Turecki G. A systematic review of association studies investigating genes coding for serotonin receptors and the serotonin transporter: I. Affective disorders. *Mol Psychiatry.* 2003, 8(6):574-91.  Furlong RA, Ho L, Walsh C, Rubinsztein JS, Jain S, Paykel ES, Easton DF, Rubinsztein DC. Analysis and meta-analysis of two serotonin transporter gene polymorphisms in bipolar and unipolar affective disorders*. Am J Med Genet.* 1998, 7;81(1):58-63.  Clarke H, Flint J, Attwood AS, Munafò MR. Association of the 5- HTTLPR genotype and unipolar depression: a meta-analysis. *Psychol Med.* 2010, 40(11):1767-78.  Risch N, Herrell R, Lehner T, Liang KY, Eaves L, Hoh J, Griem A, Kovacs M, Ott J, Merikangas KR. Interaction between the serotonin transporter gene (5-HTTLPR), stressful life events, and risk of depression: a meta-analysis. *JAMA.* 2009, 17;301(23):2462-71.  Munafò MR, Durrant C, Lewis G, Flint J. Gene X environment interactions at the serotonin transporter locus. *Biol Psychiatry.* 2009, 1;65(3):211-9.  Lasky-Su JA, Faraone SV, Glatt SJ, Tsuang MT. Meta-analysis of the association between two polymorphisms in the serotonin transporter gene and affective disorders. *Am J Med Genet B Neuropsychiatr Genet.* 2005, 133B(1):110-5.  Lotrich FE, Pollock BG. Meta-analysis of serotonin transporter polymorphisms and affective disorders. *Psychiatr Genet.* 2004, 14(3):121-9. | Met inclusion criteria but were not in the ‘5 most recent category’ (as specified in the protocol) (N=9) |
| **Gene-stress interaction** |  |
| Pinto RQ, Soares I, Carvalho-Correia E, Mesquita AR. Gene-environment interactions in psychopathology throughout early childhood: a systematic review. *Psychiatr Genet.* 2015, 25(6):223-33. | Examined only a sub-type of depression (N=1) |
| Risch N, Herrell R, Lehner T, Liang KY, Eaves L, Hoh J, Griem A, Kovacs M, Ott J, Merikangas KR. Interaction between the serotonin transporter gene (5-HTTLPR), stressful life events, and risk of depression: a meta-analysis. *JAMA.* 2009, 17;301(23):2462-71.  Munafò MR, Durrant C, Lewis G, Flint J. Gene X environment interactions at the serotonin transporter locus. *Biol Psychiatry.* 2009, 1;65(3):211-9. | Met inclusion criteria but were not in the ‘5 most recent category’ (as specified in the protocol) (N=2) |

**Table S3:** Item-by-item quality rating using the AMSTAR-2

| **AMSTAR-2 question^*^** | **1** | **2** | **3** | **4** | **5** | **6** | **7** | **8** | **9** | **10** | **11** | **12** | **13** | **14** | **15** | **16** | **Total score**  **(% satisfactory – max score: 21)** |
| --- | --- | --- | --- | --- | --- | --- | --- | --- | --- | --- | --- | --- | --- | --- | --- | --- | --- |
| **Study** |  |  |  |  |  |  |  |  |  |  |  |  |  |  |  |  |  |
| **Serotonin and 5HIAA** |  |  |  |  |  |  |  |  |  |  |  |  |  |  |  |  |  |
| Pech et al, 2018 | 1 | 0 | 1 | 0 | 0 | 0 | 0 | 1 | 1 | 0 | 1 | 0 | 1 | 1 | 0 | 1 | 8 (38%) |
| Ogawa et al, 2018 | 1 | 0 | 1 | 0 | 0 | 0 | 0 | 1 | 2 | 0 | 1 | 0 | 1 | 1 | 1 | 1 | 10 (48%) |
| Huang et al., 2020† | N/A‡ | 0 | 0 | 1 | N/A‡ | 1 | N/A‡ | 2 | 1 | 1 | 1 | 1 | 1 | 0 | N/A‡ | 1 | 9/15 (60%) |
| **Receptors and**  **Serotonin transporter (SERT)** |  |  |  |  |  |  |  |  |  |  |  |  |  |  |  |  |  |
| Nikolaus et al, 2016 | 1 | 0 | 1 | 0 | 0 | 0 | 0 | 2 | 0 | 0 | 0 | 0 | 0 | 0 | 0 | 0 | 4 (19%) |
| Wang et al., 2016 | 1 | 0 | 1 | 0 | 1 | 1 | 0 | 2 | 1 | 0 | 1 | 0 | 0 | 1 | 1 | 1 | 11 (52%) |
| Kambeitz & Howes., 2015 | 1 | 0 | 1 | 0 | 0 | 1 | 0 | 1 | 1 | 0 | 1 | 0 | 1 | 1 | 1 | 1 | 10 (48%) |
| Gryglewski et al., 2014 | 1 | 0 | 1 | 1 | 0 | 0 | 0 | 1 | 1 | 0 | 1 | 1 | 1 | 1 | 1 | 0 | 10 (48%) |
| **Depletion studies** |  |  |  |  |  |  |  |  |  |  |  |  |  |  |  |  |  |
| Ruhe et al., 2007 | 1 | 0 | 1 | 1 | 1 | 0 | 1 | 2 | 2 | 0 | 1 | 1 | 1 | 1 | 1 | 1 | 15 (71%) |
| Fusar-Poli et al, 2006 | 1 | 0 | 1 | 1 | 0 | 0 | 0 | 1 | 0 | 0 | N/A§ | N/A§ | 0 | 0 | N/A§ | 0 | 4/18 (22%) |
| **SERT gene and gene-stress interactions** |  |  |  |  |  |  |  |  |  |  |  |  |  |  |  |  |  |
| Culverhouse et al., 2018† | 1 | 2 | 1 | 2 | N/A‡ | 1 | 2 | 2 | 2 | 1 | 1 | 1 | 1 | 1 | 1 | 1 | 20/20 (100%) |
| Karg et al 2011 | 1 | 0 | 1 | 0 | 0 | 1 | 0 | 1 | 0 | 0 | 0 | 0 | 0 | 0 | 1 | 1 | 6 (29%) |
| Sharpley et al 2014 | 1 | 0 | 1 | 0 | 0 | 1 | 0 | 1 | 2 | 0 | 0 | 1 | 1 | 1 | 1 | 0 | 10 (48%) |
| Bleys et al 2017 | 1 | 0 | 1 | 1 | 0 | 0 | 0 | 1 | 1 | 0 | 0 | 0 | 0 | 1 | 1 | 0 | 7 (33%) |
| Kiyohara & Yoshimasu, 2010 | 1 | 1 | 1 | 1 | 0 | 0 | 0 | 2 | 2 | 0 | 1 | 1 | 0 | 1 | 1 | 0 | 12 (57%) |
| Oo et al., 2016 | 1 | 2 | 1 | 0 | 1 | 1 | 0 | 2 | 1 | 0 | 1 | 0 | 0 | 1 | 1 | 1 | 13 (62%) |
| Gatt et al, 2015 | 1 | 0 | 1 | 0 | 0 | 0 | 0 | 0 | 0 | 0 | N/A§ | N/A§ | 0 | 0 | N/A§ | 1 | 3/18 (17%) |
| **AMSTAR-2 question^*^** | **1** | **2** | **3** | **4** | **5** | **6** | **7** | **8** | **9** | **10** | **11** | **12** | **13** | **14** | **15** | **16** | **Total score**  **(% satisfactory – max score: 21)** |

**^*^ AMSTAR-2 questions:**

1. Did the research questions and inclusion criteria for the review include the components of PICO? (Yes: 1/No: 0).

2. Did the report of the review contain an explicit statement that the review methods were established prior to the conduct of the review and did the report justify any significant deviations from the protocol? (Yes: 2/Partial Yes: 1/No: 0).

3. Did the review authors explain their selection of the study designs for inclusion in the review? (Yes: 1/No: 0).

4. Did the review authors use a comprehensive literature search strategy? (Yes: 2/Partial Yes: 1/No: 0).

5. Did the review authors perform study selection in duplicate? (Yes: 1/No: 0).

6. Did the review authors perform data extraction in duplicate? (Yes: 1/No: 0).

7. Did the review authors provide a list of excluded studies and justify the exclusions? (Yes: 2/Partial Yes: 1/No: 0).

8. Did the review authors describe the included studies in adequate detail? (Yes: 2/Partial Yes: 1/No: 0).

9. Did the review authors use a satisfactory technique for assessing the risk of bias (RoB) in individual studies that were included in the review? (Yes: 2/Partial Yes: 1/No: 0).

10. Did the review authors report on the sources of funding for the studies included in the review? (Yes: 1/No: 0).

11. If meta-analysis was performed did the review authors use appropriate methods for statistical combination of results? (Yes: 1/No: 0).

12. If meta-analysis was performed, did the review authors assess the potential impact of RoB in individual studies on the results of the meta-analysis or other evidence synthesis? (Yes: 1/No: 0).

13. Did the review authors account for RoB in individual studies when interpreting/ discussing the results of the review? (Yes: 1/No: 0).

14. Did the review authors provide a satisfactory explanation for, and discussion of, any heterogeneity observed in the results of the review? (Yes: 1/No: 0).

15. If they performed quantitative synthesis did the review authors carry out an adequate investigation of publication bias (small study bias) and discuss its likely impact on the results of the review? (Yes: 1/No: 0).

16. Did the review authors report any potential sources of conflict of interest, including any funding they received for conducting the review? (Yes: 1/No: 0).

† **AMSTAR-2 question modifications for Huang et al. (2020) and Culverhouse et al (2018)**

3. Did the review authors explain their selection of the study designs for inclusion in the meta-analysis? (Yes: 1/No: 0).

4. Did the authors use a comprehensive search for all the relevant data (Yes: 2/Partial Yes: 1/No: 0).

6. Did the authors have access to original data? (Yes: 1/No: 0).

9. Did the authors give satisfactory consideration of potential confounders (age, race, medication use, including antidepressants, co-morbidities) (Yes: 2/Partial Yes: 1/No: 0).

12. Did the authors analyse effects of potential confounders (Yes: 1/No: 0).

13. Did the authors discuss effects of confounders? (Yes: 1/No: 0).

14. Did the review authors provide a satisfactory explanation for, and discussion of, any heterogeneity observed in the results of the synthesis? (Yes: 1/No: 0).

‡ Not applicable as the study did not conduct a systematic review.

§ Not applicable as the study did not conduct a meta-analysis.

**Table S4: item-by-item quality rating using STREGA**

| STREGA  Question | 1  a,b | 2 | 3 | 4 | 5 | 6a | 7  a,b | 8  a,b | 9  a,b | 10 | 11 | 12  a-h | 13  a-c | 14  a,b | 15 | 16  a,b,  d | 17  a-c | 18 | 19 | 20 | 21 | 22 | % satisfac-  tory (max score: 41) |
| --- | --- | --- | --- | --- | --- | --- | --- | --- | --- | --- | --- | --- | --- | --- | --- | --- | --- | --- | --- | --- | --- | --- | --- |
| Border et al 2019 | 2 | 1 | 1 | 1 | 1 | 1 | 2 | 2 | 1 | 1 | 1 | 9 | 1 | 1 | 0 | 3 | 3 | 1 | 1 | 1 | 1 | 1 | 88% |

Note. the STREGA was too long to be copied here, it can be found at: Little et al., 2009^1^

**Further detail for methods**

Algorithm for modified GRADE:

GRADE ratings are acknowledged to be inherently subjective, but with an aim to provide a reproducible and transparent framework for grading certainty of evidence.^2^ We divided certainty into high, moderate, low and very low categories. Following the approach of Kennis et al (2020)^3^ we selected criteria of relevance to the domains of interest. As many studies in these domains have been criticised as false positives due to small sample sizes,^4,5^ sample size has been prioritised by using this as a principal determinant: studies that achieve a ‘1’ start in the high category, those with a ‘0’ start in the low category. All other ‘0’ criteria move certainty down one level.

**References**

1 Little J, Higgins JPT, Ioannidis JPA, *et al.* STrengthening the REporting of Genetic Association Studies (STREGA)— An Extension of the STROBE Statement. *PLOS Med* 2009; **6**: e1000022.

2 Mustafa RA, Santesso N, Brozek J, *et al.* The GRADE approach is reproducible in assessing the quality of evidence of quantitative evidence syntheses. *J Clin Epidemiol* 2013; **66**: 735–6.

3 Kennis M, Gerritsen L, van Dalen M, Williams A, Cuijpers P, Bockting C. Prospective biomarkers of major depressive disorder: a systematic review and meta-analysis. *Mol Psychiatry* 2020; **25**: 321–38.

4 Border R, Johnson EC, Evans LM, *et al.* No support for historical candidate gene or candidate gene-by-interaction hypotheses for major depression across multiple large samples. *Am J Psychiatry* 2019; **176**: 376–87.

5 Culverhouse RC, Saccone NL, Horton AC, *et al.* Collaborative meta-analysis finds no evidence of a strong interaction between stress and 5-HTTLPR genotype contributing to the development of depression. *Mol Psychiatry* 2018; **23**: 133–42.
